# Single-incision laparoscopic surgery with transabdominal postperitoneal approach for synchronous ileocecal and sigmoid cancers: a case report

**DOI:** 10.3389/fonc.2026.1715781

**Published:** 2026-02-03

**Authors:** Junyi Li, Ruijie Lin, Jin Tang, Pengcheng Ye, Qijun Lv, Shoujiang Wei

**Affiliations:** 1 Department of Gastrointestinal Surgery, the Affiliated Hospital of North Sichuan Medical College, Nanchong, China; 2 Sichuan Clinical Research Center for Digestive Diseases, the Affiliated Hospital of North Sichuan Medical College, Nanchong, China; 3 Biotechnology Innovation Drug Application and Transformation Key Laboratory of Sichuan Province, Nanchong, China

**Keywords:** case report, colon cancer, single-incision laparoscopic surgery, synchronous multiple primary colorectal cancer, transabdominal postperitoneal approach

## Abstract

Surgical operation is the most commonly used treatment for colorectal cancer, and the treatment of synchronous multiple primary colorectal cancer is also mainly based on surgical operation. Retroperitoneal Approach for total colectomy in minimally invasive treatment of synchronous multiple colon cancer has reported. But concurrent multi-segmental resection for multiple primary colorectal cancers via a single-incision laparoscopic surgery with transabdominal postperitoneal approach has not previously been reported. We would like to share a case of radical resection of ileocecal cancer and sigmoid colon cancer via transabdominal postperitoneal approach under single-incision laparoscopy. A 72-year-old female presented to the clinic with a progressive alteration in bowel habits and stool consistency over the past year, accompanied by fatigue during the preceding month. The diagnoses included ileocecal cancer, sigmoid colon cancer, severe anemia, coronary atherosclerosis and incomplete intestinal obstruction. Blood transfusion was administered to correct the anemic condition. Given the patient’s comorbidities, including severe anemia and coronary atherosclerosis, along with a body mass index of 20.8 kg/m², a single-incision laparoscopic surgery with transabdominal postperitoneal approach was selected.

## Introduction

Colorectal cancer (CRC) ranks as the second most common cause of cancer-related mortality worldwide, representing a significant burden on global public health (34). A key clinical consideration in the management of CRC is the presence of synchronous multiple primary colorectal cancers (SMPCC), defined as the simultaneous occurrence of two or more histologically distinct malignant tumors within the colon and rectum. SMPCC accounts for approximately 1.1% to 8.0% of all primary CRC cases (19, 20, 23, 24, 26). Surgical resection remains the mainstay of curative treatment for SMPCC. Minimally invasive techniques, including laparoscopic and laparoscopic-assisted colectomy, have become the standard of care in colorectal surgery, demonstrating superior short-term and comparable long-term oncologic outcomes, as well as improved recovery profiles, when compared to open surgical approaches. Since the first reported case of single-incision laparoscopic right colectomy in 2008 (28), single-incision laparoscopic surgery (SILS) has increasingly been adopted for colorectal cancer resection. The advantages of SILS over conventional laparoscopic surgery include improved cosmetic outcomes, reduced postoperative pain, faster recovery, and greater patient satisfaction (3, 4, 12, 25, 27, 31). Previous studies have demonstrated that SILS for colorectal cancer achieves oncologic outcomes comparable to those of multiport laparoscopic surgery, both in the long-term, as well as favorable short-term perioperative results (4, 12, 18, 31, 32). Given the unique clinical features of synchronous multiple primary colorectal cancers (SMPCC), achieving minimally invasive, function-preserving, and oncologically radical resection of multiple lesions within a single operative session remains a considerable challenge for the majority of patients. This case report illustrates the successful implementation of single-incision laparoscopic surgery for synchronous ileocecal cancer and sigmoid colon cancer via transabdominal postperitoneal approach.

## Case presentation

**Figure 1:**
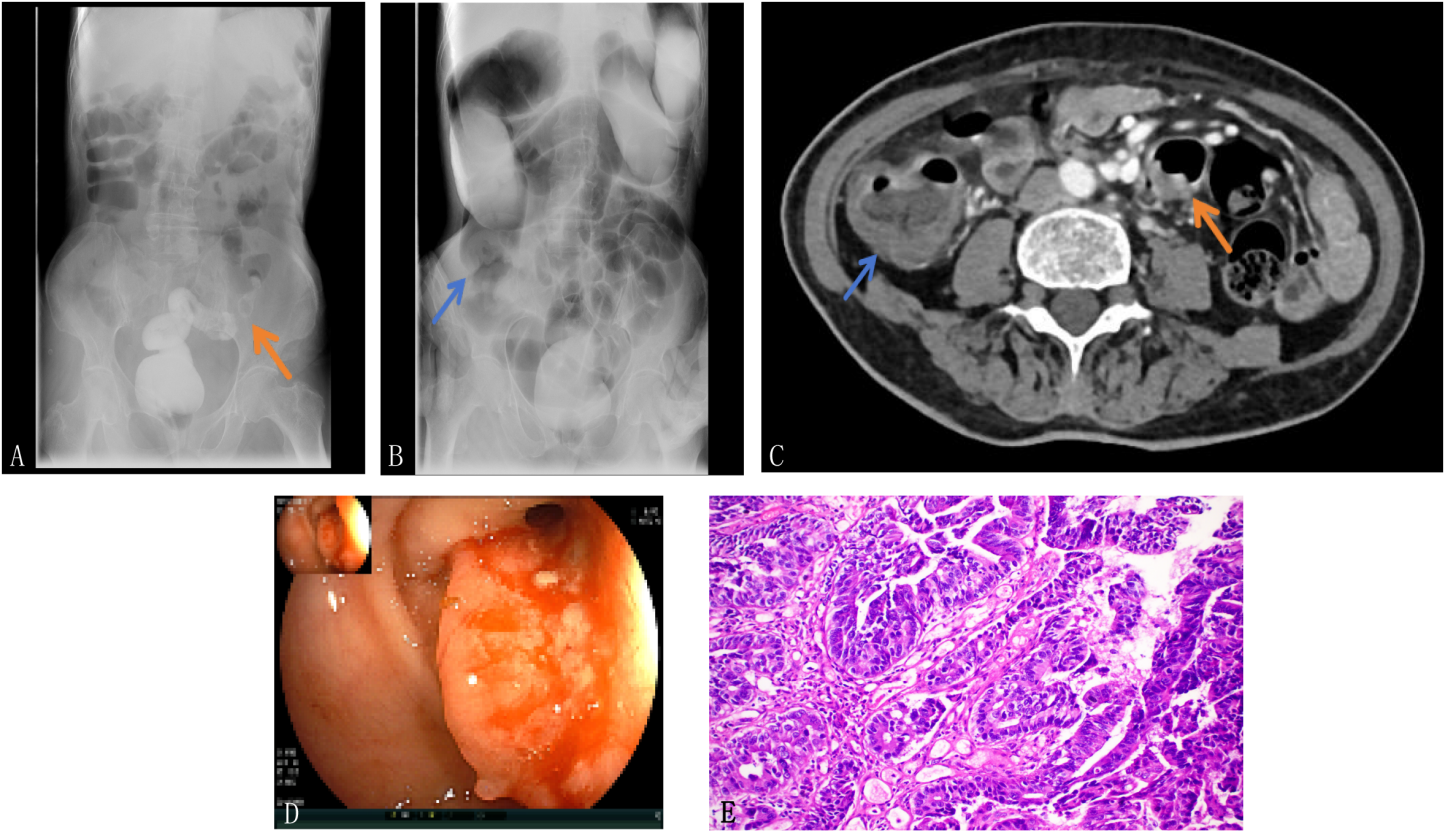
Preoperative examination. **(A)** Colonography revealed irregular filling defects in the sigmoid colon (→); **(B)** Colonography revealed irregular filling defects in the ileocecal region (→); **(C)** Abdominal CT scan with infiltrative mass and circumferential thickening in ascending colon and sigmoid colon (→); **(D)** Abdominal CT scan revealed an infiltrative mass with circumferential wall thickening in the ascending and sigmoid colon; **(E)** Biopsy examination revealed adenocarcinoma in the sigmoid colon lesion; Tissue slides: HE×20.

A 72-year-old female presented to the clinic with a progressive alteration in bowel habits and stool consistency over the past year, accompanied by fatigue over the preceding month. The patient was treated in our hospital. Physical examination revealed that the patient’s body mass index was 20.8 kg/m². Contrast-enhanced computed tomography (CT), colonoscopy, colonic contrast imaging, and histopathological verification revealed ileocecal tumor (T4aN2M0, Stage III), and sigmoid colon cancer (T3N0M0, Stage II) (**Figure 1**). Blood routine examination indicated severe anemia. Coronary angiography confirmed the presence of coronary atherosclerosis.

The ileocecal tumor resulted in anemia, while the sigmoid colon tumor caused intestinal stenosis, preventing successful passage of the colonoscope. The patient has a comorbid diagnosis of coronary atherosclerosis and is classified as ASA class IV. Based on findings from colonoscopy, abdominal CT, and colonic contrast imaging, the patient was diagnosed with SMPCC. Given the presence of anemia, bowel obstruction, and confirmed malignant neoplasms, surgical intervention is indicated to address both the symptomatic anemia and mechanical obstruction attributable to the tumors. Considering the patient’s tumor characteristics and associated complications, the intervention was designed to enhance postoperative quality of life and ensure treatment efficacy, a concurrent multi-segmental colectomy was performed as the definitive surgical strategy for radical tumor resection. Lowering the pressure of carbon dioxide insufflation can decrease the risk of exacerbating patients’ preexisting chronic conditions. Additionally, with a BMI of 20.8 kg/m^2^, the transabdominal postperitoneal approach facilitates R0 resection while decreasing the technical complexity of mesocolon mobilization and enabling more extensive lymph node dissection. Taking all these factors into consideration, the retroperitoneal approach was ultimately selected.

After obtaining the patient’s full informed consent, a single-incision laparoscopic transabdominal retroperitoneal approach was performed on April 1, 2025, involving radical resection of the sigmoid colon cancer and right hemicolectomy for the ileocecal tumor.

### Surgical technique

The patient was positioned in the supine position. Make a 3.5 cm midline incision 2 cm below the umbilicus. Inserted the single-incision device (Beijing Hang TianKaDi Technology R&D Institute, HK-60/70-60/100(0), HK-TH-60.41R) and performed abdominal exploration. Under laparoscopic guidance, the posterior peritoneum between the bilateral iliac vessels was dissected. The retroperitoneal space between Toldt’s fascia and Gerota’s fascia is then dissected until the posterior peritoneum can be elevated to the anterior abdominal wall under laparoscopic visualization. The pneumoperitoneum is subsequently closed, and removed the single-incision device, and the peritoneum was sutured to the anterior abdominal wall at the site of the single incision to create a peritoneal dome. The single-incision device was then reinserted. Retroperitoneal operative space can be established by insufflating carbon dioxide (8 mmHg) through a single-incision system.

For sigmoidectomy, the postperitoneal space was accessed along the iliac vessels and abdominal aorta using an ultrasonic dissector. Dissection was carried out in cranial, medial, and caudal directions to expand the operative field. The dissection extended craniolaterally to the inferior margin of the splenic flexure and the transition zone between the visceral and parietal peritoneum. Critical anatomical structures, including the left gonadal vessels and ureter, were identified and preserved. The origin of the inferior mesenteric artery (IMA) was exposed, followed by skeletonization of the IMA, division of the left colic artery (LCA), and ligation of the inferior mesenteric vein (IMV). The rectal mesentery was dissected to a level 5 cm distal to the tumor.

**Figure 2:**
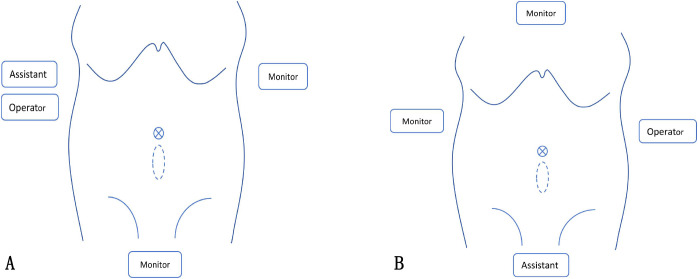
**(A)** The positions for sigmoidectomy; **(B)** The positions for right hemicolectomy.

For right hemicolectomy, the operator repositioned to the patient’s right side (**Figure 2**). Postperitoneal dissection was carried out using an ultrasonic dissector and electrosurgical hook, extending cranially to the head of the pancreas, laterally to the transition zone between the visceral and parietal peritoneum, and medially to the superior mesenteric vessels.

The ileocolic artery and vein were ligated and transected at their origins. The venous trunk of Henle was identified, and its jejunal and ileal branches were clipped and divided. The right mesocolon was completely mobilized, marking the completion of the postperitoneal phase.

The single-incision instrument was removed, the sutures anchoring the incision to the peritoneum were released, and the instrument was reinserted into the original incision. A 5 mm auxiliary port was inserted at McBurney’s point. The mesentery was incised at the level of the superior mesenteric artery (SMA) and extended caudally to the terminal ileum. A systematic lymphadenectomy was conducted along the superior mesenteric artery (SMA) and superior mesenteric vein (SMV). The right colic ligament was mobilized beyond the epiploic vascular arcade, and station VI lymph nodes were resected en bloc. The hepatocolic ligament and right parietal peritoneum were transected.

The peritoneal incision was extended inferiorly along the initial postperitoneal plane to a point 5 cm below the tumor. The left paracolic peritoneum was incised, and dissection was carried out 10 cm proximally and distally relative to the tumor. The mobilized colon, along with its mesentery and associated lymph nodes, was exteriorized through the single-incision incision. The ileum was transected 15 cm proximal to the ileocecal valve using a linear stapling device. The colon was divided 10 cm distal to the tumor. A side-to-side ileocolonic anastomosis was created using a linear stapler. The sigmoid tumor was resected with 10 cm proximal and 5 cm distal resection margins, followed by a side-to-side colorectal anastomosis performed with a linear stapler. A pelvic drainage tube was placed in the right pelvic cavity, and the abdominal component of the procedure was completed. The main surgical steps and control points are presented in the **Supplementary Video S1**.

## Results

**Figure 3:**
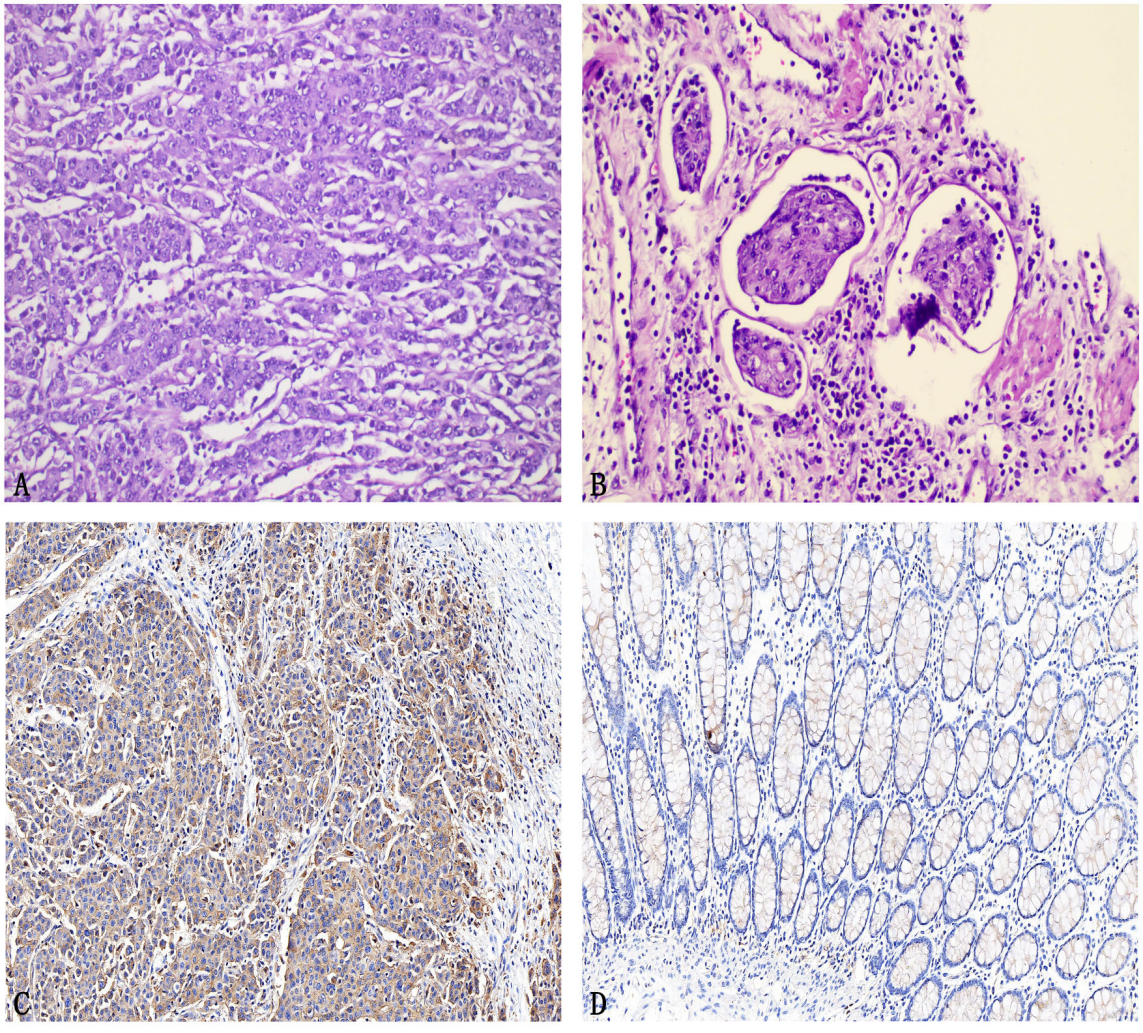
Post operative examination. **(A)** The representative image of the postoperative pathological examination findings for the ileocecal tumor (poorly differentiated adenocarcinoma), Tissue slides: HE×20; **(B)** The representative image of the postoperative pathological examination findings for the sigmoid colon tumor (moderately differentiated adenocarcinoma), Tissue slides: HE×40; **(C)** Postoperative Immunohistochemical Ki-67 Findings in Ileocecal Cancer, Tissue slides: HE×20; **(D)** Postoperative Immunohistochemical Ki-67 Findings in Sigmoid Colon Cancer, Tissue slides: HE×20.

The dissection of the specimen and ligation of the central vessels were both performed in the postperitoneal space. The duration of the surgery was 275 min, with the postperitoneal step lasting 140 min. Total blood loss was 50 ml (**Figure 3**). The relevant immunohistochemical examinations reveals low-grade adenocarcinoma in the ileocecal lesion, with evidence of intravascular and intralymphatic tumor emboli. And the tumor located in the sigmoid colon is identified as a moderately differentiated adenocarcinoma (**Figure 3**). Metastases were observed in 0 of the 17 harvested lymph nodes. All resection margins are histologically negative. The final pathological staging for both tumors is pT3N0M0, corresponding to stage IIA disease according to the AJCC TNM classification. Based on postoperative pathological findings, abdominal computed tomography, colonoscopy, and colonic contrast imaging, the diagnosis of SMPCC) was confirmed. The patient experienced flatus passage on the 2th postoperative day and resumed a liquid diet on the 4th postoperative day. The postoperative pain intensity was low, and recovery was uneventful. The patient was discharged on postoperative day 8. Nine months after the surgery, the patient’s follow-up examination results indicated that the tumor had not recurred, and at the same time, the patient’s anemia symptoms had been corrected compared to before the surgery.

## Discussion

The SMPCC represents a rare but clinically significant condition (16). Although surgical resection remains the primary treatment modality, the most appropriate surgical approach—whether multi-segment resection or total/subtotal colectomy—continues to be a topic of ongoing clinical discussion (1, 14). For patients with SMPCC, a multi-segmental colectomy approach not only reduces operative risks but also preserves colonic absorptive capacity and reservoir function, potentially improving postoperative quality of life and functional outcomes (13, 17). From a prognostic perspective, the survival and recurrence rates of patients with SMPCC are primarily influenced by the pathological features of the tumors and their clinical stage at the time of diagnosis (21).

Laparoscopic surgery has been widely recognized as a safe and effective approach for colorectal cancer resection, with short- and long-term outcomes comparable to those of open surgery (11, 29, 30, 33, 35). As surgical expertise has advanced, laparoscopic techniques have become the standard of care for colorectal cancer management at our institution. Notably, single-incision laparoscopic surgery (SILS) has emerged as a feasible alternative to conventional laparoscopic surgery (CLS), demonstrating equivalent safety profiles and oncological outcomes in both short-term and long-term follow-ups (12, 22, 32). There have been published reports on the use of SILS technology for multisegmental resection in the treatment of SMPCC (15). A comparative analysis of the short-term outcomes following simultaneous multi-segment resection using the SILS-TAPA and SILS techniques in patients with SMPCC was performed. In this case, the SILS-TAPA technique was utilized, with a surgical duration of 275 minutes, dissection of 17 lymph nodes, total blood loss of 50 mL, an 8-day postoperative hospital stay, and an uneventful recovery. The corresponding data for procedures performed using the SILS technique are as follows: the median operative time was 270 minutes, the median number of harvested lymph nodes was 26.5 for right-sided cancers (range: 8–44) and 14 for left-sided or rectal cancers (range: 3–44), and the median blood loss was 70 mL (range: 10-260). Most patients were discharged on postoperative day 10 (range: 10–20). With regard to postoperative complications, one patient developed pneumonia.

The published study has demonstrated that elevated carbon dioxide insufflation can reduce cardiac output, shorten the ventricular diastolic phase, impair pulmonary compensatory capacity, and increase the diffusion of carbon dioxide into the blood and tissues, leading to impaired gas exchange (2). The primary advantage of the retroperitoneal approach is that it avoids extensive carbon dioxide insufflation, thereby reducing the risks of exacerbation of the patient’s pre-existing chronic conditions. Additionally, in patients with a relatively lower body mass index, the retroperitoneal approach is more conducive to achieving R0 resection, while simultaneously reducing the complexity of mesocolic dissection and allowing for a more extensive lymph node dissection.

The retroperitoneal approach utilized in this study represents a significant advancement in current techniques for colorectal cancer resection. Efetov et al. previously demonstrated the feasibility of the transabdominal postperitoneal approach (5, 7–10). Efetov’s reported technique avoids entry into the abdominal cavity until the retroperitoneal space has been established. All surgical interventions in the retroperitoneal region are performed extraperitoneally. This approach is analogous to totally extraperitoneal (TEP) hernioplasty. In the establishment of the extraperitoneal space using this surgical technique, a 4 cm incision is made the anterior superior iliac spine unilaterally. Dissect through the subcutaneous tissue, aponeurosis, abdominal musculature, and transversalis fascia to reach the preperitoneal fat. The blunt dissection technique is then employed to separate the dorsal aspect of the parietal peritoneum from the anterior abdominal wall, thereby establishing the retroperitoneal anatomical space (6, 8). Our approach is technically similar to transabdominal preperitoneal laparoscopic herniorrhaphyb (TAPP). Our approach utilizes the bifurcation of the iliac vessels as a key anatomical landmark within the abdominal cavity. After entering the peritoneal cavity, the ability to access the correct surgical plane is improved, resulting in enhanced procedural feasibility. Regarding the application of the retroperitoneal approach in colorectal cancer, we have performed 36 procedures. The median total operative time in our series is 118.75 minutes, compared to 205 minutes reported by their group (10). From a numerical standpoint, our approach may offer an advantage in terms of procedural duration. With respect to surgical complexity, this technique involves lower technical demands. The current number of cases remains limited; therefore, expansion of the patient cohort is necessary, and prospective clinical studies should be conducted to validate its benefits and limitations. However, for patients with extensive intra-abdominal adhesions, Efetov’s technique may offer certain advantages.

This approach provides multiple significant advantages compared to conventional techniques. Firstly, the capacity to create the postperitoneal space under continuous laparoscopic visualization markedly improves procedural safety by minimizing the risk of unintended organ injury. Secondly, the technique adheres closely to established surgical principles, thereby enhancing its accessibility for surgeons who have undergone standard laparoscopic training. Thirdly, the simplified anatomical references and standardized procedural steps contribute to a more rapid learning curve when compared to earlier transabdominal postperitoneal approach.

The technical refinements outlined in this study address several limitations inherent in earlier transabdominal postperitoneal approach while maintaining the associated advantages, such as reduced peritoneal trauma and the potential for accelerated postoperative recovery. These advancements may broaden the clinical applicability of postperitoneal techniques in colorectal surgery, especially in cases where conventional transperitoneal access presents technical challenges. However, this is a single case report and therefore has inherent limitations. For example, the number of representative cases is limited, and the therapeutic efficacy of this approach in obese patients remains uncertain. To comprehensively evaluate the safety, feasibility, and clinical efficacy of the treatment method, a large-scale cohort study is required to further investigate the outcomes of managing SMPCC via the retroperitoneal approach.

## Conclusion

The single-incision laparoscopic surgery with transabdominal postperitoneal approach appears to be applicable for minimally invasive multi-segmental colectomy in the surgical management of synchronous multiple primary colorectal cancers.

## Data Availability

The original contributions presented in the study are included in the article/**Supplementary Material**. Further inquiries can be directed to the corresponding authors.

## References

[1] Bardakcioglu O Ahmed S . Single incision laparoscopic total abdominal colectomy with ileorectal anastomosis for synchronous colon cancer. Tech Coloproctol. (2010) 14:257–61. doi: 10.1007/s10151-010-0589-9, PMID: 20502930

[2] Casati A Comotti L Tommasino C Leggieri C Bignami E Tarantino F . Effects of pneumoperitoneum and reverse Trendelenburg position on cardiopulmonary function in morbidly obese patients receiving laparoscopic gastric banding. Eur J Anaesthesiol. (2000) 17:300–5. doi: 10.1046/j.1365-2346.2000.00662.x, PMID: 10926070

[3] Champagne BJ Papaconstantinou HT Parmar SS Nagle DA Young-Fadok TM Lee EC . Single-incision versus standard multiport laparoscopic colectomy: a multicenter, case-controlled comparison. Ann Surg. (2012) 255:66–9. doi: 10.1097/SLA.0b013e3182378442, PMID: 22104563

[4] Dong B Luo Z Lu J Yang Y Song Y Cao J . Single-incision laparoscopic versus conventional laparoscopic right colectomy: A systematic review and meta-analysis. Int J Surg. (2018) 55:31–8. doi: 10.1016/j.ijsu.2018.05.013, PMID: 29777881

[5] Efetov SK Picciariello A Tulina IA Sidorova LV Kochneva KA Bergamaschi R . Three-plane model to standardize laparoscopic right hemicolectomy with extended D3 lymph node dissection. Surg Technol Int. (2020) 36:136–42., PMID: 31821523

[6] Efetov SK Rouzy N Ghomsheei H Panova PD . Retroperitoneal approach for right hemicolectomy: two faces of one technique. Surg Endosc. (2025) 39:8748–53. doi: 10.1007/s00464-025-12367-z, PMID: 41198925

[7] Efetov SK Rychkova AK Krasnov YP . Retroperitoneal approach to D3-lymph node dissection with left colic artery preservation in the treatment of sigmoid cancer. Dis Colon Rectum. (2024). 67:e1754-5. doi: 10.1097/dcr.0000000000003354, PMID: 39268965

[8] Efetov SK Semchenko BS Rychkova AK Panova PD . A new technique of primary retroperitoneal approach for minimally invasive surgical treatment of cecal colon cancer with d3 lymph node dissection. Tech Coloproctol. (2024) 28:144. doi: 10.1007/s10151-024-03023-0, PMID: 39476294

[9] Efetov SK Zubayraeva AA Panova PD . The retroperitoneal approach to vessel-sparing D3 lymph node dissection in left-sided colorectal cancer resections: a video vignette. Colorectal Dis. (2023) 25:1940–1. doi: 10.1111/codi.16705, PMID: 37553825

[10] Efetov SK Zubayraeva AA Semchenko BS Panova PD Volgin MV Rychkova AK . Primary retroperitoneal approach for vessel-sparing D3-lymph node dissection in left colonic and rectal cancer resections - the first Russian experience. Khirurgiia (Mosk). (2023) 12:26–33. doi: 10.17116/hirurgia202312126, PMID: 38088838

[11] Fukuoka E Matsuda T Hasegawa H Yamashita K Arimoto A Takiguchi G . Laparoscopic vs open surgery for colorectal cancer patients with high American Society of Anesthesiologists classes. Asian J Endosc Surg. (2020) 13:336–42. doi: 10.1111/ases.12766, PMID: 31852023

[12] Gu C Wu Q Zhang X Wei M Wang Z . Single-incision versus conventional multiport laparoscopic surgery for colorectal cancer: a meta-analysis of randomized controlled trials and propensity-score matched studies. Int J Colorectal Dis. (2021) 36:1407–19. doi: 10.1007/s00384-021-03918-6, PMID: 33829313

[13] He W Zheng C Wang Y Dan J Zhu M Wei M . Prognosis of synchronous colorectal carcinoma compared to solitary colorectal carcinoma: a matched pair analysis. Eur J Gastroenterol Hepatol. (2019) 31:1489–95. doi: 10.1097/meg.0000000000001487, PMID: 31441800 PMC6844654

[14] Hirano Y Hattori M Sato Y Maeda K Douden K Hashizume Y . Concurrent single-incision laparoscopic right hemicolectomy and sigmoidectomy for synchronous carcinoma: report of a case. Indian J Surg. (2013) 75:293–5. doi: 10.1007/s12262-012-0696-0, PMID: 24426595 PMC3693301

[15] Ishiyama Y Hirano Y Hattori M Douden K Hashizume Y . Single incision laparoscopic surgery for multiple colorectal cancers. Asian J Endosc Surgery. (2015) 9:21–3. doi: 10.1111/ases.12245, PMID: 26487591

[16] Jesinghaus M Pfarr N Kloor M Endris V Tavernar L Muckenhuber A . Genetic heterogeneity in synchronous colorectal cancers impacts genotyping approaches and therapeutic strategies. Genes Chromosomes Cancer. (2016) 55:268–77. doi: 10.1002/gcc.22330, PMID: 26650777

[17] Jiang X Xu C Tang D Wang D . Laparoscopic subtotal colectomy for synchronous triple colorectal cancer: A case report. Oncol Lett. (2016) 12:1525–8. doi: 10.3892/ol.2016.4803, PMID: 27446464 PMC4950764

[18] Katsuno G Fukunaga M Nagakari K Yoshikawa S Azuma D Kohama S . Short-term and long-term outcomes of single-incision versus multi-incision laparoscopic resection for colorectal cancer: a propensity-score-matched analysis of 214 cases. Surg Endosc. (2016) 30:1317–25. doi: 10.1007/s00464-015-4371-y, PMID: 26139507

[19] Lam AK Carmichael R Gertraud Buettner P Gopalan V Ho YH Siu S . Clinicopathological significance of synchronous carcinoma in colorectal cancer. Am J Surg. (2011) 202:39–44. doi: 10.1016/j.amjsurg.2010.05.012, PMID: 21600553

[20] Lam AK Chan SS Leung M . Synchronous colorectal cancer: clinical, pathological and molecular implications. World J Gastroenterol. (2014) 20:6815–20. doi: 10.3748/wjg.v20.i22.6815, PMID: 24944471 PMC4051920

[21] Latournerie M Jooste V Cottet V Lepage C Faivre J Bouvier AM . Epidemiology and prognosis of synchronous colorectal cancers. Br J Surg. (2008) 95:1528–33. doi: 10.1002/bjs.6382, PMID: 18991301

[22] Liu X Li JB Shi G Guo R Zhang R . Systematic review of single-incision versus conventional multiport laparoscopic surgery for sigmoid colon and rectal cancer. World J Surg Oncol. (2018) 16:220. doi: 10.1186/s12957-018-1521-4, PMID: 30414613 PMC6230377

[23] Malapelle U De Stefano A Carlomagno C Bellevicine C Troncone G . Next-generation sequencing in the genomic profiling of synchronous colonic carcinomas: comment on Li et al. (2015). J Clin Pathol. (2015) 68:946–7. doi: 10.1136/jclinpath-2015-203205, PMID: 26139632

[24] Malesci A Basso G Bianchi P Fini L Grizzi F Celesti G . Molecular heterogeneity and prognostic implications of synchronous advanced colorectal neoplasia. Br J Cancer. (2014) 110:1228–35. doi: 10.1038/bjc.2013.827, PMID: 24434431 PMC3950856

[25] Papaconstantinou HT Thomas JS . Single-incision laparoscopic colectomy for cancer: assessment of oncologic resection and short-term outcomes in a case-matched comparison with standard laparoscopy. Surgery. (2011) 150:820–7. doi: 10.1016/j.surg.2011.07.060, PMID: 22000196

[26] Papadopoulos V Michalopoulos A Basdanis G Papapolychroniadis K Paramythiotis D Fotiadis P . Synchronous and metachronous colorectal carcinoma. Tech Coloproctol. (2004) 8 Suppl 1:s97–s100. doi: 10.1007/s10151-004-0124-y, PMID: 15655657

[27] Poon JT Cheung CW Fan JK Lo OS Law WL . Single-incision versus conventional laparoscopic colectomy for colonic neoplasm: a randomized, controlled trial. Surg Endosc. (2012) 26:2729–34. doi: 10.1007/s00464-012-2262-z, PMID: 22538676

[28] Remzi FH Kirat HT Kaouk JH Geisler DP . Single-port laparoscopy in colorectal surgery. Colorectal Dis. (2008) 10:823–6. doi: 10.1111/j.1463-1318.2008.01660.x, PMID: 18684153

[29] Song XJ Liu ZL Zeng R Ye W Liu CW . A meta-analysis of laparoscopic surgery versus conventional open surgery in the treatment of colorectal cancer. Med (Baltimore). (2019) 98:e15347. doi: 10.1097/md.0000000000015347, PMID: 31027112 PMC6831213

[30] Suda K Shimizu T Ishizuka M Miyashita S Niki M Shibuya N . Laparoscopic surgery reduced frequency of postoperative small bowel obstruction, and hospital stay compared with open surgery in a cohort of patients with colorectal cancer: a propensity score matching analysis. Surg Endosc. (2022) 36:8790–6. doi: 10.1007/s00464-022-09302-x, PMID: 35556165

[31] Takemasa I Uemura M Nishimura J Mizushima T Yamamoto H Ikeda M . Feasibility of single-site laparoscopic colectomy with complete mesocolic excision for colon cancer: a prospective case-control comparison. Surg Endosc. (2014) 28:1110–8. doi: 10.1007/s00464-013-3284-x, PMID: 24202709 PMC3973946

[32] Tei M Wakasugi M Akamatsu H . Comparison of perioperative and short-term oncological outcomes after single- or multiport surgery for colorectal cancer. Colorectal Dis. (2015) 17:O141–7. doi: 10.1111/codi.12986, PMID: 25939822

[33] Udayasiri DK Skandarajah A Hayes IP . Laparoscopic compared with open resection for colorectal cancer and long-term incidence of adhesional intestinal obstruction and incisional hernia: A systematic review and meta-analysis. Dis Colon Rectum. (2020) 63:101–12. doi: 10.1097/dcr.0000000000001540, PMID: 31804272

[34] van Rooijen S Carli F Dalton S Thomas G Bojesen R Le Guen M . Multimodal prehabilitation in colorectal cancer patients to improve functional capacity and reduce postoperative complications: the first international randomized controlled trial for multimodal prehabilitation. BMC Cancer. (2019) 19:98. doi: 10.1186/s12885-018-5232-6, PMID: 30670009 PMC6341758

[35] Zhou MW Gu XD Xiang JB Chen ZY . Clinical safety and outcomes of laparoscopic surgery versus open surgery for palliative resection of primary tumors in patients with stage IV colorectal cancer: a meta-analysis. Surg Endosc. (2016) 30:1902–10. doi: 10.1007/s00464-015-4409-1, PMID: 26281903

